# Bedside ultrasound diagnosis of atraumatic bladder rupture in an alcohol-intoxicated patient: a case report

**DOI:** 10.1186/2036-7902-4-9

**Published:** 2012-05-15

**Authors:** Michael C Daignault, Turandot Saul, Resa E Lewiss

**Affiliations:** 1 Department of Emergency Medicine, Emergency Ultrasound Division, St. Luke's-Roosevelt Hospital Center, New York, NY 10019, USA; 2 Department of Emergency Medicine, Emergency Medicine Residency Program, Lincoln Medical & Mental Health Center, Bronx, NY 10451, USA

**Keywords:** Bedside ultrasound, Bladder rupture, Alcohol intoxication, Emergency department

## Abstract

Most commonly, patients who present to the emergency department with a history and physical examination suggestive of urinary bladder rupture report a preceding traumatic event. Spontaneous atraumatic bladder rupture is relatively uncommon, but can occur in the context of a recent alcohol binge. The alcohol-intoxicated patient presents diagnostic and therapeutic challenges to the emergency physician (EP) that take on additional urgency given the high mortality of unrecognized bladder rupture. This case report reviews bladder anatomy, the unique physiological changes in the alcohol-intoxicated patient, and the high mortality rate of a ruptured urinary bladder. We review the historical diagnostic imaging options followed by a discussion of how bedside ultrasound could expedite diagnosis and management. We present the case of a patient with spontaneous atraumatic rupture of the urinary bladder after a recent alcohol binge. Bedside ultrasound was utilized by the EP to determine the need for emergent surgical consultation and intervention. We recommend that EPs consider bladder rupture in their initial evaluation of patients presenting with nonspecific abdominal pain in the context of recent alcohol intoxication. When using bedside ultrasound to evaluate the pelvis, the presence of anterior or posterior vesicular fluid collections, the loss of normal pelvic landmarks, or irregularities in the bladder wall may increase the EPs suspicion for this disease entity and expedite time-sensitive management.

## Background

The alcohol-intoxicated patient presents diagnostic and therapeutic challenges to the emergency physician (EP). Patients may be unable to provide a clear history and may present with nonspecific abdominal complaints that can mask an underlying disease process ranging in severity from gastritis to peritonitis. Patients with a history of recent heavy alcohol consumption may also present later in the disease course for a variety of reasons. Since high rates of mortality have been reported for cases of bladder rupture not recognized and managed early [1-3], bedside ultrasound may be useful in expediting the diagnosis and treatment of this surgical emergency.

## Case presentation

An 18-year-old male presented to the emergency department (ED) with several episodes of vomiting bright red blood and abdominal pain. He reported drinking tequila to the point of losing consciousness the previous night but denied daily drinking or the use of other recreational drugs. The symptoms began that morning when he awoke, about 1 h prior to presentation. He had blood-tinged emesis on his face and clothing and was noted to be pale and weak. The patient was born in Guatemala and denied any contributory medical, surgical, or medication history. On review of systems, the patient denied any recent trauma or falls. He denied fever, chills, diarrhea, blood in the stool, or history of gastrointestinal bleeding.

At triage the vital signs were as follows: blood pressure, 106/63 mmHg; heart rate, 116 beats/min; respiratory rate, 22 breaths/min; and temperature (oral), 98.2°F. The patient was ill-appearing, grimacing in pain, and clutching his abdomen. No scleral icterus was appreciated. There was no obvious abdominal deformity or sign of trauma. Hypoactive bowel sounds were appreciated on auscultation. The abdomen was diffusely tender to palpation and more focally in the epigastrium and suprapubic areas. There was mild distension and tympany in the suprapubic area. The patient had no guarding, no rebound tenderness, and no tenderness at the costovertebral angles. No blood was appreciated at the urethral meatus. The genitourinary examination was otherwise unremarkable. Examination of the skin revealed no hematomas, abrasions, or lacerations.

An intravenous line was placed, normal saline fluid was given, and blood was sent for laboratory analysis. The patient had a white blood cell count of 20.4 × 10^3^/μL (normal 4.5-10.8 × 10^3^/μL) with neutrophils 90.3% (normal 36% to 66%), hemoglobin 12.1 g/dL (normal 13.5-17.5 g/dL), hematocrit 36% (normal 41% to 53%), BUN 32 mg/dL (normal 9–20 mg/dL), creatinine 2.50 mg/dL (normal 0.66-1.25 mg/dL), potassium 4.3 mmoL/L (normal 3.5-5.1 mmoL/L), and CO_2_ 13 mmoL/L with an anion gap of 26 mmoL/L. Urinalysis demonstrated large blood and protein, and positive nitrites. An 18 French nonweighted nasogastric tube was passed without difficulty, and gastric lavage revealed a small amount of blood clots before running clear aspirates that smelled of alcohol. At this time, the patient began to complain of increased lower abdominal distension and the inability to void. A 14 French Foley catheter was inserted without difficulty and yielded 400 cc of bright red bloody urine.

**Figure 1:**
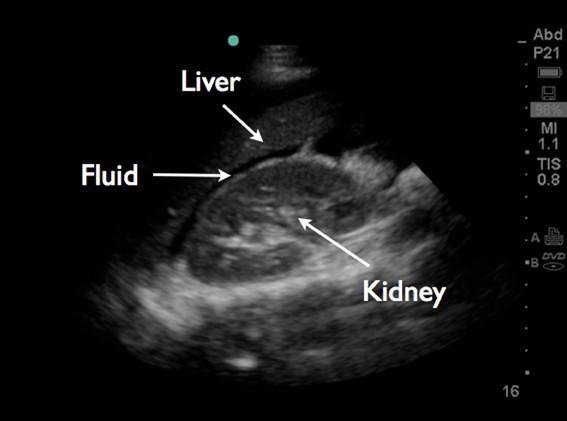
Right upper quadrant Morison's pouch view demonstrates free fluid between the liver and the right kidney.

**Figure 2:**
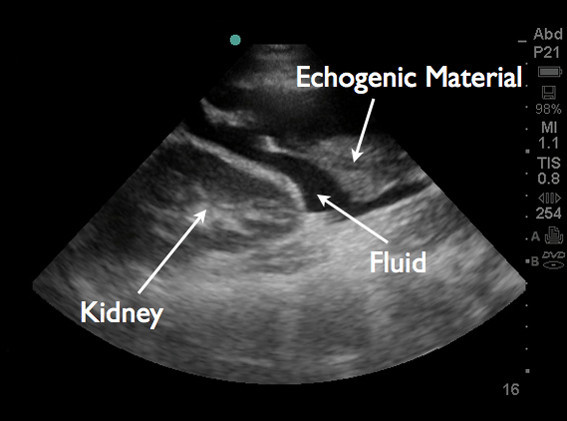
**Left upper quadrant view reveals fluid surrounding the inferior pole of the left kidney.** Echogenic material is seen floating in the fluid at the lower pole of the kidney.

**Figure 3:**
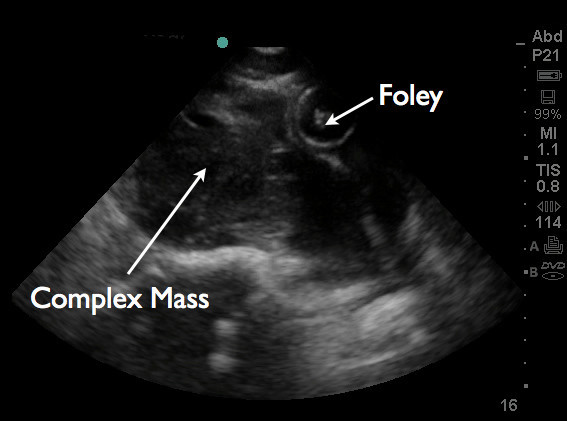
**Pelvic sagittal view.** A Foley balloon is seen as well as a heterogenous echogenic collection posterior to it. The walls of the bladder cannot be clearly delineated.

An emergent bedside ultrasound was performed by the EP using a Sonosite M-turbo ultrasound machine with a P21x/5-1 MHz phased array probe (Sonosite, Inc., Bothell, WA, 98021, USA). The right upper quadrant Morison's pouch view revealed free fluid between the liver and the right kidney (Figure 1). The left upper quadrant view revealed fluid between the left kidney and spleen. At the lower pole of the kidney, echogenic material was found to be floating in anechoic fluid (Figure 2). The pelvic view revealed a Foley catheter balloon with loss of anatomic landmarks to determine its exact location. There was anechoic free fluid in the rectovesicular pouch. The bladder walls could not be clearly delineated, and a large complex mass of heterogeneous echogenicity was seen posteriorly, thought to represent clotted blood (Figure 3). The patient continued to complain of pain despite narcotic pain medication. On repeat abdominal examination, the patient had developed rebound tenderness and guarding.

**Figure 4:**
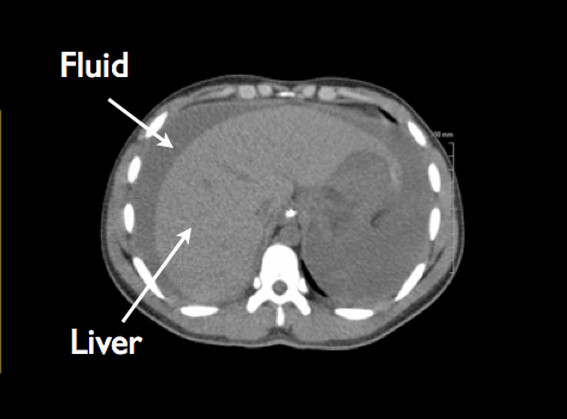
CT of the abdomen showing extensive free fluid.

**Figure 5:**
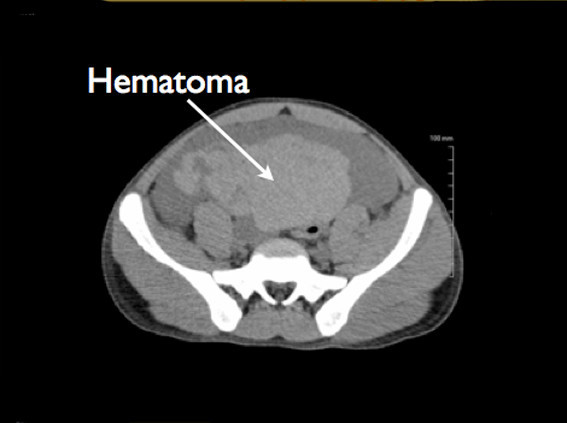
A large high-density collection in the inferior abdomen compatible with a hematoma.

**Figure 6:**
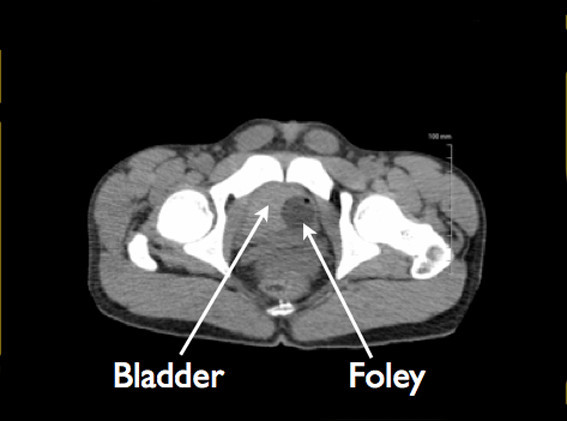
**Collapsed urinary bladder.** The urinary bladder is relatively collapsed around a Foley catheter with high-density fluid within the bladder compatible with blood products.

The urology service was emergently consulted, and computerized tomography (CT) scan of the abdomen and pelvis was immediately performed without contrast given patient's elevated serum creatinine. The CT scan confirmed extensive abdominal free fluid (Figure 4), and a large high-density collection in the inferior abdomen compatible with a hematoma measuring 11.2 × 9 × 7.6 cm (Figure 5). The urinary bladder was collapsed around a Foley catheter with high-density fluid within the bladder compatible with blood products (Figure 6). These findings were consistent with rupture of the urinary bladder.

The patient was taken emergently to the operating room. Antibiotics were administered and blood was typed and crossed. Laparotomy confirmed the diagnosis and revealed a 3.5-cm burst-type laceration at the bladder dome with actively bleeding edges and clot in the bladder. The edges were trimmed, and the defect was repaired with two layers of sutures. The following morning, laboratory analysis revealed a white blood cell count of 7.6 × 10^3^/μL, hemoglobin 8.3 g/dL, hematocrit 23.5, BUN 11, and creatinine 0.60 mg/dL. The recovery was unremarkable, and the patient was discharged on postoperative day 3.

## Discussion

The empty, or contracted, urinary bladder lies entirely within the bony pelvis and is considered extraperitoneal. The full bladder extends above the protection of the pelvic inlet into the peritoneum to about the level of the umbilicus. The bladder dome, also the most mobile part of the bladder, is particularly susceptible to injury at its posterior-superior extension point [1]. The rupture mechanism has been described as a burst rupture due to increased intrabladder pressure. The dome is unique in its isolated peritoneal reflection so that rupture in this area most likely results in intraperitonal urinary contamination [4].

In the emergency setting, urinary bladder rupture can be a complication of both blunt and penetrating trauma, or can occur spontaneously in both the diseased and nondiseased bladder. Some authors have postulated that an increasing number of patients are presenting with spontaneous rupture in nondiseased bladders in the context of a recent alcohol binge, further classified as a subset of idiopathic bladder ruptures [5]. The alcohol-intoxicated patient may have decreased sensation of and abnormal behavioral response to bladder filling, leading to an increased risk of bladder rupture. The sheer volume of ingested alcohol and its diuretic effect increase bladder filling, often to the point of extensive distension. This distention may lead to an atonic decompensated bladder that is so stretched and thinned that even minor trauma may cause it to rupture [6].

`Most cases of spontaneous bladder rupture in association with alcohol consumption can have a positive outcome if they are recognized and treated early. Extraperitoneal ruptures, in particular, can be managed conservatively. However, intraperitoneal ruptures at the bladder dome always require surgical intervention. High mortality rates have been previously reported in the literature. As early as 1959, Bastable et al. reported a series of cases of bladder rupture associated with alcohol intoxication that had a mortality rate of 50% [2]. Festini et al. cited a mortality rate of 12% for spontaneous intraperitoneal bladder ruptures in the context of an alcohol binge [1]. In 2005, Sezhian et al. reported a 22% mortality rate for patients with traumatic bladder rupture after blunt trauma [3]. Schneider reported a high mortality rate of 22% to 44% with traumatic bladder rupture but attributed the high rate to severe mechanisms of injury and associated head, abdominal, and pelvic trauma [7]. Some authors have speculated that the high mortality rate could also be attributed to a delay in diagnosis and intervention that results in serious complication from lack of control of urine extravasation [3]. This can result in intra-abdominal and pelvic abscesses, sepsis, and metabolic derangements. Significant peritoneal reabsorption of urea and creatinine can masquerade as acute renal failure on initial biochemical testing, as observed in our patient. Electrolyte abnormalities such as hyperkalemia, hypernatremia, uremia, or acidosis may also occur as extravasated urine is reabsorbed in the peritoneal cavity. Electrocardiogram abnormalities resulting from hyperkalemia may be seen. Several authors have noted increased mortality rates after severe symptom onset [3,6].

EPs should consider this diagnosis in patients presenting with nonspecific worsening abdominal pain in the context of a recent alcohol binge. Maddocks and Leadbetter recognized in 1976 that ‘bladder rupture is seldom considered in the differential diagnosis of peritonitis’ [8]. A patient who presents after a known mechanism of injury, such as motor vehicle collision or other abdominopelvic trauma, will raise the EP's suspicion for bladder rupture, especially in context of a pelvic fracture. In patients who are unable to provide a history regarding the previous night's events or do not have obvious signs of injury, the diagnosis may be delayed or missed. According to Herd et al., alcohol intoxication increases the risk of sustaining minor causative trauma that may be forgotten, leading to an erroneous diagnosis of atraumatic rupture or failure to suspect the injury at all [6]. These patients may also present, after trying to self-medicate, a perceived ‘worse-than-average’ hangover with antacids and painkillers, a greasy meal, or other hangover remedies. Since these patients present in a delayed fashion, they may rapidly deteriorate in the ED.

Historically, imaging modalities to evaluate a patient with suspected bladder injury have included retrograde cystography, CT retrograde cystography, and delayed CT cystography. The advantages and disadvantages of each have been well discussed elsewhere [5,9,10]. Research indicates that even when retrograde cystography is strongly indicated, it is rarely performed because it is both resource and time consuming [9], factors that may have also contributed to historically high mortality rates for bladder rupture. Meanwhile, the combined abdominopelvic CT exam has become the modality of choice for EPs navigating nonspecific complaints. Conventional abdominal noncontrast CT has an accuracy rate of only 60.6% for bladder injury compared to 95.9% for the retrograde cystogram [9]. However, in a patient with worsening abdominal pain/distention and vital sign abnormalities, a CT scan will likely be the imaging modality of choice

Bedside ultrasound is ideally used during the initial assessment of the patient with nonspecific abdominopelvic complaints. Although fluid collection from a suspected bladder rupture is most commonly seen collecting posterior to the bladder in a supine patient, anterior collection of fluid has also been reported [10], so the EP should always evaluate both vesicular areas. The presence and contour of the superior bladder wall can also be visualized and evaluated for irregularities.

Additional research on the sensitivity and specificity of such ultrasound findings for bladder rupture is needed. Free fluid observed in focused ultrasound views of the right and left upper quadrants, as seen in our patient, accompanied by bladder abnormalities observed in the suprapubic view, could indicate a combination of extravasated urine and blood, and further raise the EP's suspicion of bladder rupture.

## Conclusion

We recommend that EPs consider bladder rupture in their initial evaluation of nonspecific abdominopelvic pain in the context of recent alcohol binge. When performing the suprapubic view in assessment of the pelvis with ultrasound, the EP should pay particular attention to evaluating both the anterior and posterior vesicular spaces, as well as for bladder wall abnormalities and loss of normal pelvic landmarks. Bedside ultrasound cannot definitively rule out bladder rupture, but it can help the EP expedite management in this disease entity with time-sensitive high mortality rates.

## Consent

Written informed consent was obtained from the patient for publication of this case report and any accompanying images. A copy of the written consent is available for review by the Editor-in-Chief of this journal.

## Endnote

This case report has not been presented or submitted elsewhere. There is no grant support or involvement.

## Abbreviations

ED: emergency department; EP: emergency physician; CT: computerized tomography.

## Competing interests

There is no grant support or involvement. The authors declare that they have no competing interests.

## Authors' contributions

MD reviewed the current literature, drafted the manuscript, and was responsible for the case report's overall organization. Both TS and RL were involved in helping to critically revise the manuscript for important intellectual content and have given final approval of the version to be published. TS procured both the sonographic and CT images, and labeled them. RL collaborated with TS in verifying anatomic labeling of our images along with consulting a radiologist at our institution. All authors read and approved the final manuscript.
